# The role of adiponectin in ischemia-reperfusion syndrome: a literature review

**DOI:** 10.31744/einstein_journal/2020RW5160

**Published:** 2020-08-21

**Authors:** Mariela Carolina Santos Carballo, Luís Claudio Santos Pinto, Marcus Vinicius Henriques Brito

**Affiliations:** 1 Universidade do Estado do Pará BelémPA Brazil Universidade do Estado do Pará, Belém, PA, Brazil.

**Keywords:** Adiponectin, Ischemia, Reperfusion injury

## Abstract

Adiponectin, among other diverse adipokines, is produced in greater quantity and has an effect on the adipose tissue and other tissues in the body. Adiponectin plays three main roles: regulatory metabolic and sensitizing function of insulin in the liver and muscles; it acts as an anti-inflammatory cytokine and in vascular protection, besides important cardiac protection in the presence of ischemia-reperfusion syndrome. Since many situations resulting from traumatic accidents or pathologies are due to cell damage caused by ischemia-reperfusion syndrome, it is relevant to study new therapeutic alternatives that will contribute to reducing these lesions. The objective of this study is to carry out a literature review on the role of adiponectin in ischemia-reperfusion syndrome.

## INTRODUCTION

The adipose tissue produces several cytokines. Adiponectin (APN), among many other adipokines, is produced in greater quantities and has an effect on the adipose tissue and other tissues in the body. Current studies show a production of APN in other cells, in addition to adipocytes, such as macrophages, lymphocytes, endothelial and epithelial cells.^(1-5)^

Among its functions, APN has three main roles: it regulates metabolism and insulin sensitivity in the liver and muscles; acts as an anti-inflammatory cytokine and in vascular protection; and has a cardioprotective effect in the presence of ischemia- reperfusion syndrome (IRS).^(1,2,6-14)^

A significant number of articles have recently suggested the possible therapeutic uses of APN to reduce tissue damage caused by IRS in several organs. The exogenous use of this cytokine was able to reduce *in vitro* and *in vivo* apoptosis and necrosis in myocardial, brain, vascular, hepatic and renal tissues after IRS. However, the molecular details of how these protective effects of APN occur are still unclear in the literature.^(2)^

Considering that several outcomes of traumas or pathologies are consequences of cell damage caused by IRS, it is crucial that new therapy alternatives be studied to help decrease these injuries. Therefore, the objective of this study is to conduct a literature review on the role of APN in IRS (Table 1).

**Table 1:** Number of articles including the keywords “*adiponectin*”, “*ischemia*” and “*reperfusion*”, published in English and with free access to full text at the database PubMed®

	Year of publication								
	2012	2013	2014	2015	2016	2017	2018	2019	Total
Number of articles	3	8	3	8	3	9	4	1	39

## DISCUSSION

### Adiponectin and ischemia-reperfusion syndrome in the heart muscle

The pro-inflammatory states found in chronic diseases, especially those related to states of metabolic dysfunction, such as obesity and diabetes, are observable causes of hypoadiponectinemia. An important activation pathway of APN occurs through CP-3, an azapeptide from the class of selective CD36 binders, which leads to the activation of receptors activated by gamma peroxisome proliferator-activated receptors (PPARγ), one of the most important regulators of APN transcription. There are also some polymorphisms found in the APN gene that cause a decrease of its plasma levels in humans, and among them is isoleucine substituted by threonine in the position 164 (I164T). This mutation is strongly linked to the development of hypertension and coronary artery disease in individuals of different ethnic groups.^(1-5,7)^

Braun et al., described that APN deficiency increases the size of myocardial infarction after ischemic reperfusion and leads to exaggerated cardiac hypertrophy after pressure overload. These processes are causally linked to mitochondrial dysfunction, which can happen in some diseases, such as diabetes and heart failure, in which APN values are reduced.^(15,16)^

Wang et al., studied the molecular mechanisms responsible for the transmembrane signaling of APN and its cardioprotective effect. To that end, they compared wild mice to knockout mice for caveolin-3 (Cav-3KO). Caveolin acts as a potent signal inhibitor and suppressor of growth; however, some studies have suggested that, in the case of insulin, it acts as a facilitator for its action. Insulin, in turn, shares several biological functions with APN, such as glucose intake, lipid oxidation and cardiovascular protection. Therefore, caveolin facilitates insulin action which, as a consequence, activates APN through the APN receptor complex AdipoR1 with caveolin-3 (AdipoR1/Cav-3), determining that APN fulfill its anti-ischemia and cardioprotective role through AMPK (adenosine monophosphate-activated protein kinase), which was significantly higher in the group of wild mice in comparison to the group Cav-3KO.^(17,18)^

Huynh et al., evaluated the cardioprotective effects of CP-3, an azapeptide from the new class of selective CD36 binders. CD36 signalling allows the activation of the receptor and activator of peroxisome proliferation, a regulator of APN transcription. They concluded that there was an increase in APN circulating levels through CP-3.^(19)^

Another important protein involved in the mechanism against myocardial injury is the CTRP9. Kambara et al., verified the protective effect of this protein against myocardial injury after IRS in mice. CTRP9 constitutes an express protein in the adipose tissue that acts like APN, benefiting glucose metabolism and promoting endothelium-dependent vasodilation, and its expression is altered in obese and insulin-resistant individuals. Through intravenous administration of CTRP9, prior to promoting ischemia and after promoting myocardial perfusion in mice, this study confirmed a decrease in the extension of the injury caused by myocardial infarction in the animals of the study groups, through AMPK activation via the AdipoR1 receptor in the cardiac myocytes resulting from the endocrine action of CTRP9. There was also a mitigation of inflammatory cytokine expression, such as the tumor necrosis factor alpha (TNF-α) and interleukin 6 (IL-6).^(1,7,20)^

Lymphotoxin alpha, evaluated by Lau et al., in a study with mice subjected to myocardial IRS, was proven an important suppressor protein of plasma APN expression, starting 72 hours after myocardial reperfusion in animals subjected to 30 minutes of myocardial ischemia. TNF-α, an APN suppressor cytokine, also increased after reperfusion. Therefore, a therapy combining anti-TNF-α and anti-lymphotoxin alpha could restore APN serum levels in patients with hypoadiponectinemia verified in cases of IRS. Gao et al., reported that one single injection of etanercept provides cardioprotective effects by neutralizing TNF-α.^(21,22)^

Zhang et al., investigated if AdipoRon, the first orally active molecule that binds APN receptors, could protect the heart against injuries from myocardial ischemia and reperfusion. The results demonstrated that AdipoRon, an oral activator of the active APN receptor, effectively mitigated post-ischemia cardiac injury by supporting APN receptor agonists (via AMPK), and thus becoming a new and promising therapeutic approach to treat cardiovascular complications caused by disorders related to obesity, such as type 2 diabetes. Hypoadiponectinemia leads to an imbalance in the autophagic flow in diabetic individuals, which decreases the antioxidant function mediated by autophagosome clearance in the heart tissue. AdipoRon, via AMPK, stimulated the formation of these autophagosomes, increasing clearance, reducing infarction area, and improving cardiac function.^(23,24)^

On the other hand, Zhang et al., stated that APN antioxidative and anti-inflammatory role does not occur via AMPK, but via protein kinase A (PKA). The researchers suggested that, when they administered APN 10 minutes before promoting IRS in the heart muscle, there was a decrease in oxidative stress and a reduction of the infarcted area in the study group. However, these effects were not observed in knockout mice in more than 70% for PKA expression. In these animals, there was significant inhibition of the protective effects of APN in cardiomyocytes, with a reduction in the activation of the nuclear factor kappa B (NF-kB). In the group of animals with AMPK deficiency, the protective and antioxidant action of APN via PKA dependent on NF-kB inhibition was intact.^(6)^ Potenza et al., also corroborated the importance of APN via AMPK, but said that this pathway only occurs together with the signaling of the SIRT-1 pathway, in which both are responsible for regulating APN cardioprotective activity.^(25)^

Tomicek et al., showed for the first time that elderly and oophorectomized female rats and adult female rats also benefited from APN administration after IRS installation in the myocardium. However, they saw that the mechanisms through which APN determines its protective effect in the myocardium seem to occur in diverse ways and there are differences regarding the changes in the responses mediated by the pathways of phosphor-AMPK and NOX2, reinforcing the fact that adaptative responses to IRS are also influenced by the levels of circulating estrogens.^(26)^

Lin et al., hypothesized that N-acetylcysteine, with its antioxidative function, could improve or restore cardioprotection after sevoflurane conditioning in rats. This conditioning is compromised by diabetes, leading to increased oxidative stress. For the study, they used control rats and rats with type 1 diabetes induced by streptozotocin, treated or not with N-acetylcysteine for four weeks, and subjected to myocardial ischemia-reperfusion injury, in the absence or presence of sevoflurane. In this study, N-acetylcysteine, combined with post-conditioning by sevoflurane, synergistically reduced the size of the infarction in the group of diabetic rats.^(27)^

Osmotin, which is found in mammals and is homologous to APN, was used by Liu et al., and also seemed to suggest a protective effect in the myoblasts H9c2 of rats after IRS. They also proposed that this protein may have induced PI3K/AKT activation and inhibited NF-kB, leading to an inhibiting effect of cell apoptosis and of the expression of inflammatory cytokines.^(28)^

### Adiponectin and ischemia-reperfusion syndrome in the pulmonary tissue

Li et al., studied a treatment with APN which activated AMPK, increased eNOS expression and mitigated iNOS expression in rats. The results of the present study showed that APN has protective effects against ischemia–reperfusion–induced lung injury (IRLI) due to its anti-inflammatory and antioxidant effects and antiapoptotic activity. These APN protective effects were eliminated in rats with *diabetes mellitus* type 2, in which IRLI was exacerbated. The present study suggested that APN can be a potential therapeutic agent for IRLI in *diabetes mellitus* type 2.^(29)^

### Adiponectin and ischemia-reperfusion syndrome in the hepatic tissue

The improvement of damages caused by IRS and the decrease in hypoadiponectinemia were also observed by Zhang et al., when they evaluated hepatocyte function in rats submitted to liver IRS. The exogenous administration of APN via AMPK reduced the increment of glutamic oxaloacetic transaminase (GOT) and glutamic-pyruvic transaminase (GPT), the quantity of hepatic necrosis, and the inflammatory cell infiltrate. In addition, pro-inflammatory cytokines were found in relation to the Control Group.^(30)^

Xia et al., showed that the survival of rats treated with APN during ischemia and reperfusion after autologous liver transplant improved significantly in comparison to the rats that only received regular saline solution. Therefore, alterations in the circulating levels of APN can have significant long-term implications in transplants. The mechanism involved in APN protection has many factors, including anti-inflammatory and antiapoptotic properties, as shown by the decrease in production of myeloperoxidase (MPO) and inflammatory cytokines, such as TNF-α and IL-6. Adiponectin also prevents apoptosis of bile duct cells.^(31)^

### Adiponectin and ischemia and reperfusion syndrome in kidney function

Jin et al., conducted a study that allowed them to state that the genetic deficiency of APN protected the kidney of mice against acute renal injury caused by IRS. They compared the group of wild mice to the group of knockout mice for APN and saw that the latter presented lower values of serum creatinine and lower tubular damage or apoptosis after 30 minutes of renal ischemia, followed by reperfusion, than the Control Group. The reduction of apoptosis occurred through the decrease of Bax (proapoptotic protein found in epithelial cells of the renal tubule) and the diminished activity of p53 and caspase-3. There is a decrease in the infiltration of inflammatory cells and the production of pro-inflammatory molecules in the kidney; suppression of NF-kB activation and promotion of macrophage migration through the activation of kinase PI3. These results suggest that APN has a pivotal role in the pathogenesis of ischemia and reperfusion through the regulation of inflammation and apoptosis.^(3)^

According to Song et al., APN plasma levels are significantly increased in patients with renal dysfunction and are inversely related to the risk of cardiovascular mortality. First of all, the ischemia-reperfusion injury is amplified in the presence of chronic kidney failure, as shown by compromised cardiac contractile function, increased infarction size and elevated apoptosis of cardiomyocytes in a type of rat submitted to subtotal nephrectomy. The injury from ischemia and reperfusion in mice with renal failure is even more intensified in the absence of cardiac APN, and significantly improved by the exogenous supplement of the human recombinant globular domain of APN (gAD), but not full-length APN, which constitutes the first evidence of the benefits of gAD administration in cardiovascular results after kidney failure.^(32)^

### Adiponectin and ischemia-reperfusion syndrome in the central nervous system

Jung et al.,^(33)^ reported that the protective effects have propelled investigations about APN action in the cerebral vascular system. Hypoadiponectinemia would be a significant independent risk factor for cerebrovascular disease, while there would be increased risk of mortality for patients with hypoadiponectinemia who suffered ischemic insults. Moreover, patients with advanced intracranial atherosclerosis showed significantly low plasma APN levels 6 to 12 hours after ischemia. In an experimental study, these authors verified that the group of knockout rats for APN (APN-KO) showed a significant higher leukocyte adhesion than the Control Group after brain IRS. The activated leukocytes that adhered to the endothelium released toxic mediators that damaged the surrounding vasculatures or the parenchymal cells, or induced an alteration in the blood rheology and accelerated thrombosis, which resulted in platelet aggregation. The inhibition of endothelial adhesion-accumulation of leukocytes after brain IRS improved electrophysiological and neurological function, reduced cerebral edema and the size of the infarction area. Thus, APN, which impeded the leukocyte-endothelium interaction by inhibiting secondary inflammatory reaction, showed a neuroprotective property in models of ischemia-reperfusion. Adiponectin inhibits neuronal apoptosis and relieves oxidative stress in neurons submitted to IRS. The possible pathway linked to APN action in the nervous tissue is the AMPc/PKA pathway (AMPc-dependent protein kinase)-CREB (AMPc response element-binding protein) -BDNF (brain derived neurotrophic factor).^(33-35)^

Wang et al., verified that APN could mitigate oxygen and glucose deprivation in HT22 cells of the hippocampus through the signaling of the Janus kinases/signal transducer and activator of transcription proteins (JAK/STAT) pathway, protecting them from mitochondrial oxidative stress and apoptosis. Studies showed that the activated JAK2/STAT3 pathway protects against hypoxia and injury from oxygenation, decreases neurotoxicity induced by amyloid β1-42 in the SH-SY5Y glioma cells, and promotes neuroprotection and neural plasticity in models of ischemia in murine.^(36)^

Zhang et al., analyzed the use of genetic modification through cell therapy with APN in rat neurons. They concluded that the use of APN at this level improved behavioral function and density in micro vessels, reduced the infarction area, and the rate of brain cell apoptosis.^(37)^

Song et al., studied the therapeutic role of gAD (globular segment of the carboxy termination of APN, which is more potent than the protein in the whole) in ischemic brain injuries of rats with *diabetes mellitus* type 1. They defined a study through which the results showed that gAD improved the neurological scores and reduced the volume of infarction in rats with *diabetes mellitus* type 1. Thus, the interventions that reinforce the expression of AdipoR1 receptors during early stages of ischemia and gAD supplementation during advanced stages can reduce ischemic brain injuries in diabetic patients.^(38)^

### Adiponectinemia and ischemia-reperfusion syndrome in the pancreas and intestine

Du et al., studied the APN effect on the protection of transplanted pancreatic islets in mice that were damaged by IRS through nuclear activation and transcription of the COX2-TNF-α-NF-Kb pathway. They verified that APN suppressed the production of TNF-α and Ikb phosphorilation, reducing the injuries from IRS and apoptosis of the islets, in addition to improving islet function *in vitro* and *in vivo*.^(2)^

Liu et al., studied APN effects in intestinal IRS in rats. They verified that pre-treatment with recombinant APN via AMPK/HO-1 mitigated intestinal injury, reduced the production of pro-inflammatory cytokines, including IL-6, IL-1β and TNF-α, the production of MDA (malondialdehyde) was inhibited and the release of SOD (superoxide dismutase) was restored.^(39)^

**Figure 1:**
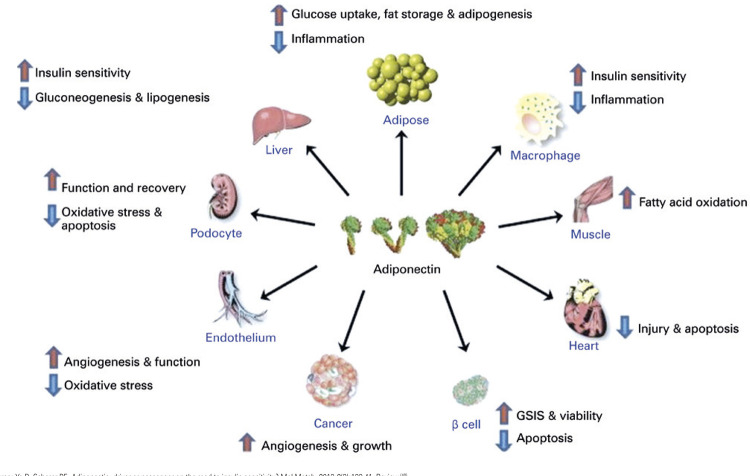
Summary overview of physiological and cell changes in response to recombinant adiponectin protein or endogenously overproduced adiponectin GSIS: Glucose-Stimulated Insulin Secretion.

Source: Ye R, Scherer PE. Adiponectin, driver or passenger on the road to insulin sensitivity? Mol Metab. 2013;2(3):133-41. Review.^(40)^

Figure 1 summarizes the important actions of APN in several target-organs.^(40)^

We can then observe the important anti-inflammatory and antiapoptotic effect of APN on injuries from IRS in several organs and systems in the more recent experimental studies. What warrants further attention and are not yet clearly defined are the signaling cascade pathways through which these protective APN effects occur, since there is still some controversy between different authors.
