# Supplementary material for: The role of adiponectin in ischemia-reperfusion syndrome: a literature review
Source: Einstein (Sao Paulo). 2020 Aug 21;18:eRW5160. doi: 10.31744/einstein_journal/2020RW5160 (PMC7444600; doi:10.31744/einstein_journal/2020RW5160)
Supplement: Translation

# Translation: O papel da adiponectina na síndrome de isquemia e reperfusão: revisão de literatura

**Authors:** Mariela Carolina Santos Carballo, Luís Claudio Santos Pinto, Marcus Vinicius Henriques Brito

**Affiliations:** 1 Brasil Universidade do Estado do Pará, Belém, PA, Brasil.

**Keywords:** Adiponectina, Isquemia, Traumatismo por reperfusão

## Abstract

A adiponectina, em meio a outras diversas adipocinas, é a produzida em maior quantidade e exerce efeitos no próprio tecido adiposo e em outros diversos tecidos do organismo. Dentre suas funções, a adiponectina apresenta três principais papéis: função metabólica regulatória e sensibilizadora da insulina no fígado e nos músculos atua como citocina anti-inflamatória e vasculoprotetora, além de exercer importante fator cardioprotetor na presença da síndrome de isquemia e reperfusão. Visto que inúmeras situações decorrentes de acidentes traumáticos ou patologias recaem no dano celular causado pela síndrome de isquemia e reperfusão, observa-se a importância de estudar novas alternativas terapêuticas que venham a contribuir para a diminuição dessas lesões. O objetivo do presente estudo é realizar uma revisão de literatura sobre o papel da adiponectina na síndrome de isquemia e reperfusão.

## INTRODUÇÃO

O tecido adiposo é responsável pela produção de inúmeras citocinas. A adiponectina (APN), em meio a outras diversas adipocinas, é a produzida em maior quantidade e exerce efeitos no próprio tecido adiposo e em outros diversos tecidos do organismo. Trabalhos atuais mostram a produção de APN também por outras células além dos adipócitos, como macrófagos, linfócitos, células endoteliais e epiteliais.^(1-5)^

Dentre suas funções, a APN apresenta três principais papéis: função metabólica regulatória e sensibilizadora da insulina no fígado e nos músculos, atua como citocina anti-inflamatória e vasculoprotetora, além de exercer importante fator cardioprotetor na presença da síndrome de isquemia e reperfusão (SIR).^(1,2,6-14)^

Recentemente, significativo número de artigos apontou para possíveis usos terapêuticos da APN na redução dos danos teciduais causados pela SIR em diversos órgãos. O uso exógeno desta citocina mostrou-se positivo em reduzir apoptose e necrose *in vitro* e *in vivo* em tecidos miocárdico, cerebral, vascular, hepático e renal após a SIR. Porém os detalhes moleculares de como esses efeitos protetores da APN acontecem ainda são incertos na literatura.^(2)^

Visto que inúmeras situações decorrentes de acidentes traumáticos ou patologias recaem no dano celular causado pela SIR, observa-se a importância de se estudar novas alternativas terapêuticas que venham a contribuir para a diminuição destas lesões. Portanto, o objetivo do presente estudo realizar uma revisão de literatura sobre o papel da APN na SIR (Tabela 1).

**Tabela 1:** Número de artigos que incluem as palavras-chave “*adiponectin*”, “*ischemia*” e “*reperfusion*” publicados em língua inglesa e com conteúdo integral de livre acesso encontrados na base de dados PubMed®

	Ano de publicação								
	2012	2013	2014	2015	2016	2017	2018	2019	Total
Número de artigos	3	8	3	8	3	9	4	1	39

## DISCUSSÃO

### Adiponectina e síndrome de isquemia e reperfusão no músculo cardíaco

Os estados pró-inflamatórios encontrados em doenças crônicas, especialmente aquelas ligadas a estados de disfunção metabólica, como a obesidade e o diabetes, são causas observáveis de hipoadiponectinemia. Uma importante via de ativação da APN ocorre por meio do CP-3, um azapeptídeo pertencente à classe de ligantes CD36 seletivos, que leva à ativação dos receptores ativados por proliferadores de peroxissoma tipo gama (PPARγ), um dos mais importantes reguladores de transcrição da APN. Existem também alguns polimorfismos encontrados no gene da APN que cursam com a diminuição de seus níveis plasmáticos em humanos, e dentre eles está a isoleucina substituída por treonina na posição 164 (I164T). Esta mutação encontra-se fortemente ligada ao desenvolvimento de hipertensão e doença arterial coronária, em indivíduos de diferentes grupos étnicos.^(1-5,7)^

Braun et al., descreveram que a deficiência de APN leva ao aumento do tamanho do infarto do miocárdio após a reperfusão isquêmica e à hipertrofia cardíaca exagerada após sobrecarga de pressão. Esses processos estão causalmente ligados à disfunção mitocondrial, que pode ocorrer em doenças como o diabetes e a insuficiencia cardíaca, nas quais os valores de APN estão diminuídos.^(15,16)^

Wang et al., estudaram os mecanismos moleculares responsáveis pela sinalização transmembrana da APN e seu efeito cardioprotetor. Para tanto, compararam camundongos tipo selvagens com camundongos *knockout* para caveolina-3 (Cav-3KO). A caveolina age como potente sinalizador inibidor e supressor de crescimento, porém estudos têm sugerido que, no caso da insulina, ela age como um facilitador para sua ação. A insulina, por sua vez, compartilha muitas funções biológicas com a APN, como a captação de glicose, a oxidação lipídica e a proteção cardiovascular. Desta forma, a caveolina facilita a ação da insulina, que, por consequência, ativa a APN, por meio do complexo do receptor para APN AdipoR1 com a caveolina-3 (AdipoR1/Cav-3), determinando que a APN exerça seu papel anti-isquêmico e cardioprotetor via AMPK (proteína quinase adenosina monofosfato ativada), o que foi significativamente maior no grupo de camundongos selvagens, quando comparados com o grupo Cav-3KO.^(17,18)^

Huynh et al., avaliaram também os efeitos cardioprotetores do CP-3, um azapeptídeo da nova classe dos ligantes seletivos do CD36. A sinalização do CD36 permite a ativação do receptor e do ativador de proliferação de peroxissomo, um regulador da transcrição de APN. Concluíram que houve aumento dos níveis circulantes de APN pelo CP-3.^(19)^

Outra proteína importante, que participa do mecanismo contra a injúria miocárdica, é a CTRP9. Kambara et al., verificaram o efeito protetor dessa proteína contra a lesão miocárdica após a SIR em camundongos. A CTRP9 constitui uma proteína expressa no tecido adiposo que age como a APN exercendo efeito benéfico no metabolismo da glicose e promovendo vasodilatação dependente do endotélio, tendo sua expressão alterada em indivíduos obesos e resistentes à insulina. Por meio de administração endovenosa de CTRP9, antes de promover a isquemia e após promover a reperfusão miocárdica em camundongos, este trabalho confirmou uma diminuição no tamanho da extensão da lesão por infarto miocárdico nos animais do grupo estudo, por meio da ativação de AMPK via receptor AdipoR1 nos miócitos cardíacos consequentes da ação endócrina da CTRP9. Também houve atenuação da expressão de citocinas inflamatórias, como fator de necrose tumoral alfa (TNF-α) e interleucina 6 (IL-6).^(1,7,20)^

A linfotoxina alfa, avaliada por Lau et al., em estudo com camundongos submetidos a SIR miocárdica, mostrou-se importante proteína supressora da expressão da APN plasmática, a partir de 72 horas após a reperfusão miocárdica em animais submetidos a 30 minutos prévios de isquemia neste órgão. O TNF-α, citocina também supressora da APN, igualmente aumentou após a reperfusão. Desta forma, uma terapia combinada com anti-TNF-α e anti-linfotoxina alfa poderia restaurar os níveis séricos de APN nos pacientes com hipoadiponectinemia verificada em ocasião de SIR. Gao et al., relataram que uma única injeção de etanercept exerce efeitos cardioprotetores neutralizando ao TNF-α.^(21,22)^

Zhang et al., investigaram se o AdipoRon, a primeira molécula ativa oralmente que liga receptores APN, poderia proteger o coração contra lesões de isquemia e reperfusão miocárdicas. Os resultados demonstraram que o AdipoRon, um ativador do receptor de APN ativo por via oral, atenuou eficazmente a lesão cardíaca pós-isquêmica, suportando os agonistas do receptor de APN (via AMPK), e tornando-se uma nova abordagem terapêutica promissora para o tratamento de complicações cardiovasculares causadas por distúrbios relacionados com a obesidade, como diabetes tipo 2. A hipoadiponectinemia leva a um desajuste no fluxo autofágico em indivíduos diabéticos, causando diminuição na função antioxidante mediada pelo *clearance* dos autofagossomos no tecido cardíaco. O AdipoRon, via AMP-K, estimulou a formação destes autofagossomos, aumentando seu *clearance*, reduzindo a área de infarto, e melhorando a função cardíaca.^(23,24)^

Por outro lado, Zhang et al., afirmaram que o papel protetor antioxidativo e anti-inflamatório da APN não ocorre via AMPK, e sim via proteinoquinase A (PKA). Os pesquisadores sugeriram que, ao administrar a APN 10 minutos antes de promover a SIR em músculo cardíaco, ocorreram, no grupo estudo, diminuições do estresse oxidativo e da área infartada. Porém, não foi possível observar estes efeitos nos camundongos *knockout* em mais de 70% para a expressão da PKA. Nesses animais, houve inibição significativa dos efeitos protetores da APN nos cardiomiócitos, com diminuição da ativação de fator nuclear kappa B (NF-kB). Já no grupo de animais com deficiência para AMPK, a ação protetora e antioxidante da APN via PKA dependente da inibição de NF-kB manteve-se intacta.^(6)^ Potenza et al., também corroboraram a importância da APN via AMPK, porém sinalizaram que esta via so acontece em conjunto com a sinalização da via do SIRT-1, na qual ambas atuam como corresponsáveis pela regulação da atividade cardioprotetora da APN.^(25)^

Tomicek et al., demonstraram pela primeira vez que ratas idosas e ooforectomizadas, assim como ratas adultas, também se beneficiaram com a administração da APN após a instalação da SIR em miocárdio. Porém, verificaram que os mecanismos pelos quais a APN determina seu efeito protetor no miocárdio parecem acontecer de formas diferentes, havendo divergências quanto às mudanças nas respostas mediadas pelas vias da fosfo-AMPK e NOX2, reafirmando que as respostas adaptativas à SIR também sofrem influência dos níveis de estrógenos circulantes.^(26)^

Lin et al., hipotetizaram que a N-acetilcisteína, com sua função antioxidante, poderia melhorar ou restaurar a cardioproteção pós-condicionamento por sevoflurano em ratos. Este condicionamento se encontra comprometido no diabetes, levando a um aumento do estresse oxidativo. Para isto, usaram ratos controles ou diabéticos de tipo 1 induzidos por estreptozotocina que foram tratados ou não com N-acetilcisteína durante 4 semanas e sujeitos à lesão de isquemia-reperfusão miocárdica, na ausência ou presença de sevoflurano. Neste estudo, a N-acetilcisteína, em conjunto com o pós-condicionamento por sevoflurano, reduziu sinergicamente o tamanho do infarto nos grupos de ratos diabéticos.^(27)^

A osmotina, um homólogo da APN encontrado em mamíferos, foi utilizada por Liu et al., e também pareceu sugerir efeito protetivo em mioblastos H9c2 de ratos após SIR. Foi sugerido que esta proteína induziu a ativação de PI3K/AKT e inibiu NF-κB, resultando em efeito inibidor da apoptose celular e da expressão de citocinas inflamatórias.^(28)^

### Adiponectina e síndrome de isquemia e reperfusão no tecido pulmonar

Li et al., estudaram um tratamento com APN, a qual ativou a AMPK, aumentou a expressão de eNOS e atenuou a expressão de iNOS em ratos. Os resultados do presente estudo demonstraram que a APN exerce efeitos protetores contra a injúria pulmonar por isquemia e reperfusão (IPIR) por meio de seu efeito anti-inflamatório, antiestresse oxidativo e atividades antiapoptóticas. Esses efeitos protetores da APN foram eliminados em ratos com *diabetes mellitus* tipo 2, nos quais a IPIR foi exacerbada. O presente estudo indicou que a APN pode ser potencial agente terapêutico para IPIR no *diabetes mellitus* tipo 2.^(29)^

### Adiponectina e síndrome de isquemia e reperfusão no tecido hepático

A melhora dos danos causados pela SIR e a diminuição da hipoadiponectinemia também foram observadas por Zhang et al., quando avaliaram a função de hepatócitos em ratos submetidos à SIR hepática. A administração exógena de APN via AMPK reduziu o incremento de transaminase glutâmico-oxalacética (TGO) e transaminase glutâmico-pirúvica (TGP), a quantidade de necrose hepática e menos infiltrado celular inflamatório, assim como citocinas pró-inflamatorias foram encontradas em relação ao Grupo Controle.^(30)^

Xia et al., mostraram que a sobrevivência de ratos tratados com APN durante a isquemia e a reperfusão subsequente no transplante de fígado autólogo melhorou significativamente em comparação com os ratos que receberam apenas solução salina normal. Assim, alterações nos níveis circulantes de APN podem ter importantes implicações no transplante a longo prazo. O mecanismo envolvido na proteção da APN é multifatorial, incluindo propriedades anti-inflamatórias e antiapoptose, conforme ilustrado pela diminuição da produção de mieloperoxidase (MPO) e das citocinas inflamatórias, como TNF-α e IL-6. A APN também evita a apoptose das células do ducto biliar.^(31)^

### Adiponectina e síndrome de isquemia e reperfusão na função renal

Jin et al., realizaram um estudo que os permitiu afirmar que a deficiência genética da APN protegeu o rim de camundongos contra lesão renal aguda causada pela SIR. Foi comparado o grupo de camundongos selvagens com o grupo de camundongos *knockout* para APN, e observou-se que este apresentou menor valor de creatinina sérica e menor dano tubular ou apoptose após 30 minutos de isquemia renal, seguido por reperfusão, que os animais do Grupo Controle. A diminuição da apoptose se deu pela diminuição da Bax (proteína pró-apoptótica encontrada nas células epiteliais do túbulo renal) e atividade reduzida de p53 e caspase-3. Há diminuição da infiltração de células inflamatórias e da produção de moléculas pró-inflamatórias no rim; supressão da ativação de NF-kB e promoção da migração de macrófagos pela ativação da quinase PI3. Estes resultados indicam que a APN desempenha papel fundamental na patogênese da isquemia e da reperfusão por meio da regulação da inflamação e da apoptose.^(3)^

Segundo Song et al., os níveis plasmáticos de APN estão significativamente aumentados em pacientes com disfunção renal e inversamente relacionados com o risco de mortalidade cardiovascular. Em primeiro lugar, a lesão por isquemia e reperfusão é amplificada na presença de insuficiência renal crônica, como evidenciado por função contrátil cardíaca comprometida, tamanho do infarto aumentado e elevação da apoptose de cardiomiócitos em um modelo de ratos submetidos à nefrectomia subtotal. A lesão por isquemia e reperfusão em camundongos com insuficiência renal é ainda mais intensificada na ausência de APN cardíaca, e melhorada acentuadamente pelo suplemento exógeno do domínio globular recombinante humano de APN (gAD), mas não APN de comprimento total, o que constitui a primeira evidência para os benefícios da administração de gAD em resultados cardiovasculares após insuficiência renal.^(32)^

### Adiponectina e síndrome de isquemia e reperfusão e sistema nervoso central

Jung et al.,^(33)^ relataram que os efeitos protetores têm impulsionado a investigação sobre as ações da APN no sistema vascular cerebral. A hipoadiponectinemia seria fator de risco independente e significativo para doença cerebrovascular, enquanto existiria um risco aumentado de mortalidade para pacientes com hipoadiponectinemia que sofreram insultos isquêmicos. Além disso, pacientes com aterosclerose intracraniana avançada tiveram seus níveis de APN plasmáticas significativamente baixos 6 e 12 horas após isquemia. Em estudo experimental, estes autores verificaram que o grupo de ratos *knockout* para APN (APN-KO) mostrou significativamente maior adesão de leucócitos do que o Grupo Controle, após SIR cerebral. Os leucócitos ativados que aderiram ao endotélio liberaram mediadores tóxicos que danificaram as vasculaturas circundantes ou as células parenquimatosas, ou induziram alteração na reologia sanguínea e trombose acelerada, que levou à agregação plaquetária. A inibição da adesão-acumulação endotelial dos leucócitos após a SIR cerebral melhora a função eletrofisiológica e neurológica, reduz o edema cerebral e o tamanho da área de enfarte. Por conseguinte, a APN, que impediu a interação de leucócitos-endotélio, inibindo a reação inflamatória secundária, mostrou-se neuroprotetora em modelos de isquemia-reperfusão. A APN inibe a apoptose neuronal e alivia o estresse oxidativo em neurônios submetidos à SIR. A possível via ligada à ação da APN no tecido nervoso é a via AMPc/PKA (*AMPc-dependent protein kinase*)-CREB (*AMPc response element-binding protein*) - BDNF (*brain derived neurotrophic factor*).^(33-35)^

Wang et al., verificaram que a APN pode atenuar a privação de oxigênio e glicose nas células HT22 do hipocampo pela sinalização da via *Janus kinases/signal transducer and activator of transcription proteins* (JAK/STAT), protegendo-as do estresse oxidativo mitocondrial e apoptose. Estudos revelaram que a via JAK2/STAT3 ativada protege contra hipóxia e lesão por oxigenação, diminui a neurotoxicidade induzida por β_1-42_ amiloide nas células SH-SY5Y do glioma, e promove neuroproteção e plasticidade neural em modelos de isquemia em murinos.^(36)^

Zhang et al., verificaram o uso de modificação genética por terapia celular com APN em neurônios em ratos. Concluíram que o uso da APN a esse nível melhorou a função comportamental e a densidade de microvasos, assim como diminuiu a área de infarto e a taxa de apoptose celular neuronal.^(37)^

Já Song et al., estudaram o papel terapêutico do gAD (segmento globular da terminação carboxi da APN, que se apresenta mais potente que a proteína como um todo) na lesão isquêmica cerebral de ratos com *diabetes mellitus* tipo 1. Definiram um estudo por meio do qual os resultados mostraram que o gAD melhorou os escores neurológicos e reduziu os volumes de infarto em ratos com *diabetes mellitus* tipo 1. Assim, as intervenções que reforçam a expressão de receptores AdipoR1 durante estádios iniciais de isquemia e suplementação de gAD durante estádios avançados podem reduzir a lesão isquêmica cerebral em pacientes diabéticos.^(38)^

### Adiponectina e síndrome de isquemia e reperfusão em pâncreas e intestino

Du et al., estudaram o efeito da APN na proteção de ilhotas pancreáticas transplantadas em camundongos que foram danificadas pela SIR por meio da ativação e transcrição nuclear da via COX2-TNF-α-NF-Kb. Verificaram que a APN suprimiu a produção do TNF-α e a fosforilação do Ikb, diminuindo as lesões pela SIR e a apoptose das ilhotas, além de melhorar a função destas tanto *in vitro* como *in vivo*.^(2)^

Liu et al., estudaram o efeito da APN na SIR intestinal em ratos. Verificaram que o pré-tratamento com APN recombinante via AMPK/HO-1 atenuou a lesão intestinal, reduziu a produção de citocinas pró-inflamatórias, incluindo IL-6, IL-1β e TNF-α, a produção de MDA (malondialdeído) foi inibida e a libertação de superóxido dismutase (SOD) foi restaurada.^(39)^

Na sequência, a figura 1 resume as importantes ações da APN em diversos órgãos-alvo.^(40)^

**Figura 1:** Resumo geral das mudanças fisiológicas e celulares em resposta à proteína adiponectina recombinante ou adiponectina superproduzida endogenamente GSIS: *Glucose-Stimulated Insulin Secretion*.

Fonte: Ye R, Scherer PE. Adiponectin, driver or passenger on the road to insulin sensitivity? Mol Metab. 2013;2(3):133-41. Review.^(40)^

Observa-se, portanto, o importante efeito anti-inflamatório e antiapoptótico da APN em ocasião de lesão por SIR em diversos órgãos e sistemas nos estudos experimentais mais recentes. O que ainda merece atenção, por não estarem bem definidas até o momento, são as vias de cascata de sinalização, pelas quais estes efeitos protetores da APN ocorrem, havendo ainda certa contradição entre os autores.
